# A Case Report of Necrotizing Fasciitis Managed With the Application of Negative Pressure Wound Therapy to Achieve Better Wound Closure

**DOI:** 10.7759/cureus.46140

**Published:** 2023-09-28

**Authors:** Maheen Afzal, Hassan Shakoor, Mohammad Talal Afzal

**Affiliations:** 1 Medicine, Jinnah Hospital Lahore/Allama Iqbal Medical College, Lahore, PAK; 2 Medicine, Fauji Foundation Hospital/Foundation University Medical College, Rawalpindi, PAK; 3 Medicine, Rawalpindi Medical University, Rawalpindi, PAK

**Keywords:** vacuum assisted closure (vac), plastic surgery, surgical debridement, methicillin resistant staphylococcus aureus (mrsa), negative pressure wound therapy, necrotizing fasciitis

## Abstract

Necrotizing fasciitis is a severe and potentially life-threatening infection of the soft tissues that involves the skin, subcutaneous fat, fascia, and muscle. It can rapidly spread and lead to tissue death, sepsis, toxic shock syndrome, cardiopulmonary failure, and even death, especially in patients with chronic diseases, immunocompromised status, or immobility. To control the spread of necrosis, prompt diagnosis and aggressive surgical intervention with radical debridement of the affected tissues are essential, along with the administration of broad-spectrum antibiotics and intensive care support, when required. The application of negative pressure wound therapy has been utilized in the management of acute and complicated wounds with good outcomes.

Here, we present a case of an 82-year-old female who presented with fever, tachycardia, and hypotension with underlying comorbid conditions of diabetes mellitus, hypertension, and spinal stenosis. On further exploration, she was found to have necrotizing fasciitis involving the left gluteal region. The present article describes the use of a vacuum-assisted closure dressing as an adjunct to serial debridement in the treatment of severe necrotizing fasciitis.

## Introduction

Necrotizing fasciitis (NF) is a serious and rare infection that affects the skin, subcutaneous fat, fascia, and muscle tissue, and can cause rapid bacterial spread along the fascial planes [1]. It is a rare disease with an annual incidence of about 1.5 cases/100,000 population, but the mortality is rather high, reaching about 20-30% [2].

Two types of NF have been identified, with type 1 being caused by multiple organisms (Streptococcal, Staphylococcal, Enterococcal, Enterobacteriaceae, and Bacteroides species) and type 2 typically involving group A Streptococci [2]. The most common causes of NF are insect bites, chronic wounds, trauma, and idiopathic origin, with rare iatrogenic sources like injections or surgical wounds [3].

Successful and prompt diagnosis through surgery is crucial in managing necrotizing fasciitis [1]. After surgical debridement and the administration of systemic antibiotics based on bacterial culture, a large open wound typically remains, which is traditionally managed using conventional dressing techniques, such as dry or wet gauze, before covering the wound with a mucocutaneous flap, skin graft, or flap. However, these conventional dressing techniques can lead to significant morbidities when handling exposed wounds [4].

In this regard, advanced wound dressings can be used as alternative treatment options to reduce pain and exudates, improve patients’ social interactions and quality of life, as well as enable early hospital discharge and early return to daily activities [4].

Negative pressure wound therapy (NPWT) or vacuum-assisted closure (VAC) dressing is one such advanced wound dressing, which was introduced commercially after studies carried out by Argenta and Morykwas in 1997 [4]. This therapy is an important adjunctive method in wound treatment, accelerating the process of repair and wound bed preparation until definitive coverage through several methods of tissue reconstruction is done [5]. Therefore, this report aims to describe the use of negative pressure wound therapy in the management of NF.

## Case presentation

Our patient, an 82-year-old female, was a diagnosed case of spinal stenosis involving the lumbar spine. She was receiving intra-articular steroid injections for the treatment of her condition. Five days before her admission, she was given an intra-muscular painkiller injection at the local dispensary.

**Figure 1:**
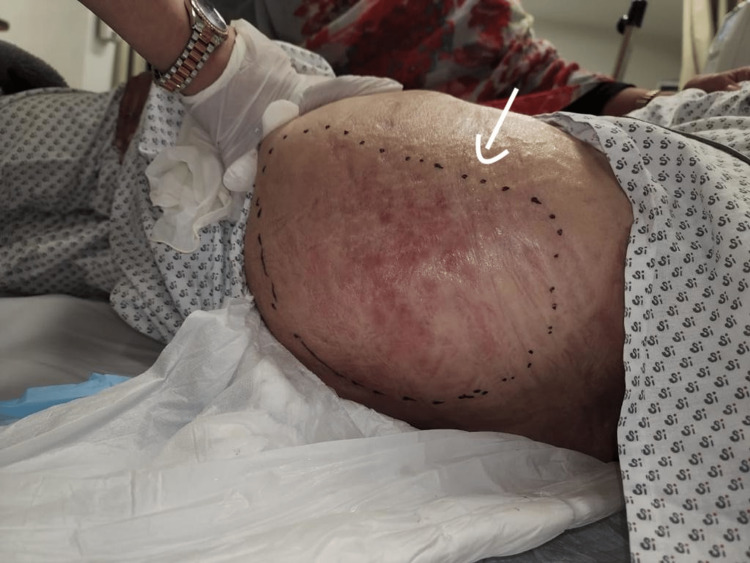
Left gluteal region on initial presentation.

She presented to the emergency department in a toxic state. At the time of presentation, she was febrile, hypotensive, tachypneic, and tachycardic. Initial vital signs were a temperature of 102.1 degrees Fahrenheit, a blood pressure of 96/61 mmHg, a pulse rate of 112 beats per minute, a respiration rate of 19 breaths per minute, and an oxygen saturation of 96% on room air. On examination of her left gluteal, warmth, redness, and swelling were noticed (Figure 1).

**Figure 2:**
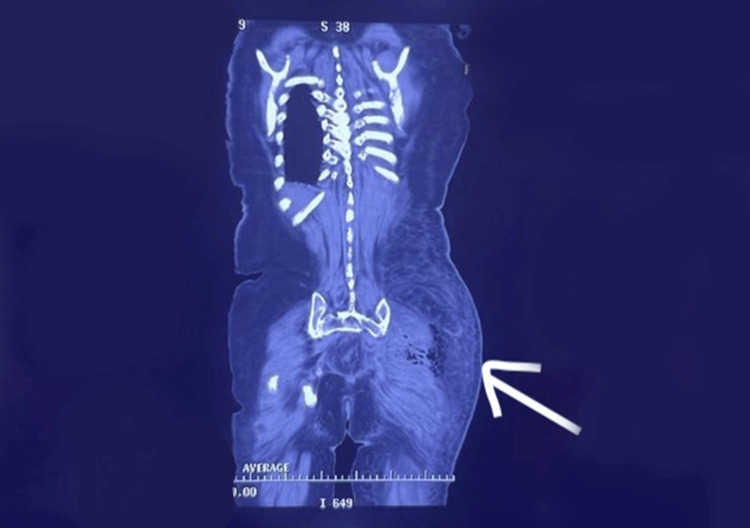
CT scan film, performed at the time of presentation

The patient was administered 1L Ringers lactate I/V and empiric broad-spectrum antibiotics (meropenem and linezolid). Relevant investigations, including an urgent non-contrast CT scan of the abdomen and pelvis, were ordered immediately, which showed: Subcutaneous soft tissue edema in the left gluteal region, left thigh, and left lateral lower abdominal wall (Figure 2); air in the left gluteal soft tissue and left gluteal maximus muscle (Figure 2); and the left gluteal maximus, medius, and left pyriformis muscle were swollen and edematous.

Urgent baseline investigations were sent when the patient presented to the emergency department (Table 1). Her Hb was 11.2 g/dL, raised WBC count (22,000), raised inflammatory markers (198 mg/dL), deranged renal function tests (RFTs) (creatinine 2.39 mg/dL), and raised creatine phosphokinase (CPK) levels (1287). Her serum electrolytes came out to be in the normal range.

**Table 1:** Baseline investigation results sent from the emergency department

Lab Test	Result
Hemoglobin	11.2 [g/dL]
White blood cells (WBC)	22,000 [WBCs per microliter]
Neutrophils	78%
C-reactive protein (CRP)	198 [mg/dL]
Creatinine	2.39 [mg/dL]
Creatine phosphokinase test (CPK)	1287 [U/L]

**Figure 3:**
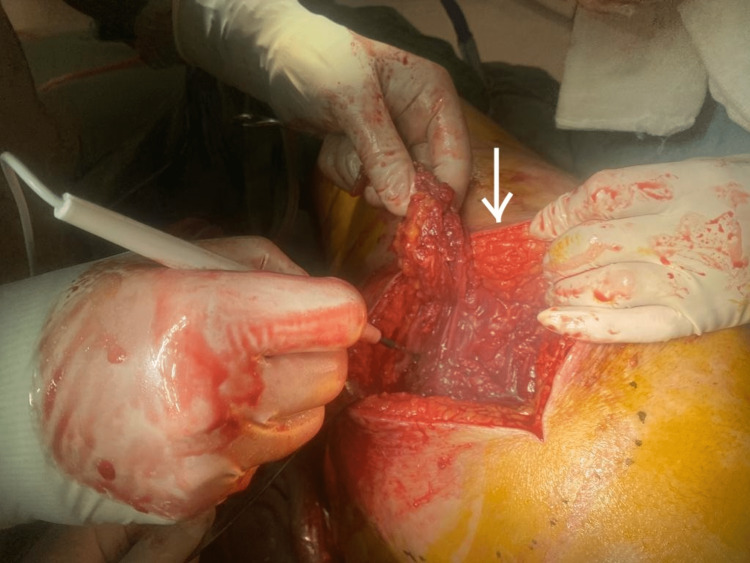
Necrotic tissue seen after the first exploration

**Figure 4:**
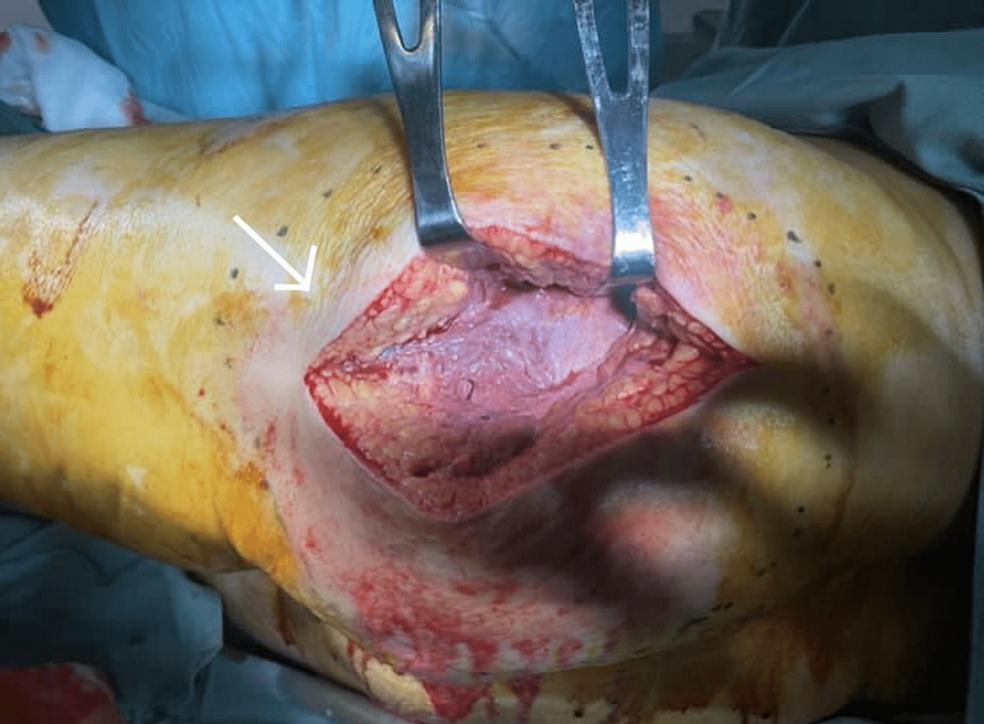
First debridement done on the day of presentation

Based on the combination of the patient's clinical symptoms, imaging, and laboratory results, it was strongly suspected that the patient had necrotizing fasciitis. A team of plastic surgeons was consulted, and the patient was posted for emergency exploration and surgical debridement. Non-viable necrotic tissue was found after she was explored for the first time (Figure 3). Debridement was done, and the necrotic tissue was removed (Figure 4).

Surgical wound cultures were sent, which yielded the growth of methicillin-resistant Staphylococcus aureus (MRSA), sensitive to linezolid. She was then admitted to the intensive care unit and was continued on meropenem and linezolid. Daily wound debridement was decided for her by the plastic surgeons till the wound was optimized and the plan for VAC to achieve speedy wound closure was postponed till her wound got better.

The patient underwent a total of six surgical debridements. Conventional dressings under aseptic measures were applied after each debridement and the peri-lesional area was cleaned daily with a 2% chlorhexidine topical solution.

**Figure 5:**
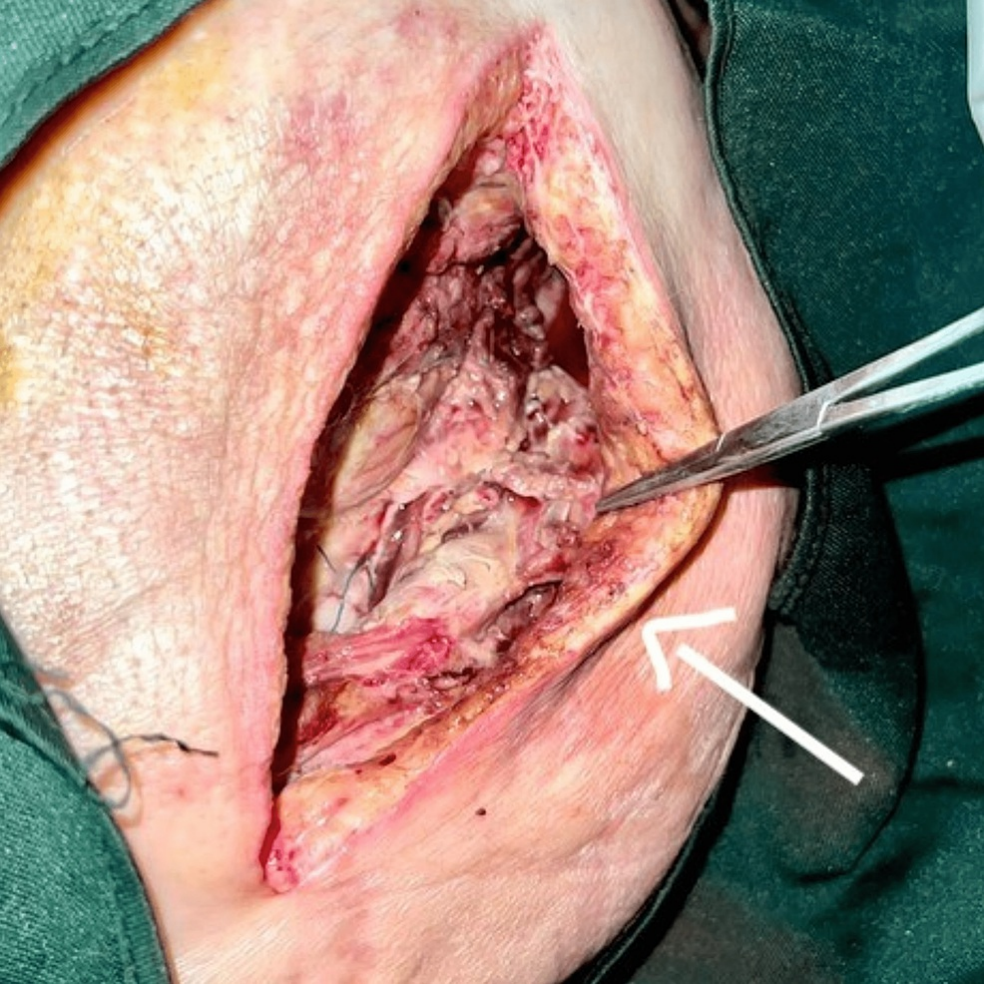
The wound after three sessions of debridement

On the third day of admission, her wound showed significant improvement with reduced swelling and redness (Figure 5). The necrotic tissue was reduced. Surgeons decided to continue the plan for daily debridement for the next few days. This was done to avoid any aggravation of infection and to keep the wound clean.

On the fourth day of admission, the patient started to improve clinically, i.e. tachycardia settled, there were no fever spikes, blood pressures were in the normal range, there was increased oral intake, improved RFTs, and declining trends of C-reactive protein (CRP) and total leucocyte count (TLC).

On the seventh day of admission, negative pressure wound therapy (VAC dressing) was applied to the gluteal region at a pressure of 125 mmHg in continuous mode. No interruption in the therapy was noticed during the hospital stay apart from changing the VAC dressing when soaked. The wound dimensions before the application of VAC dressing were 18 centimeters in length and 6 centimeters in width in the left gluteal region.

**Figure 6:**
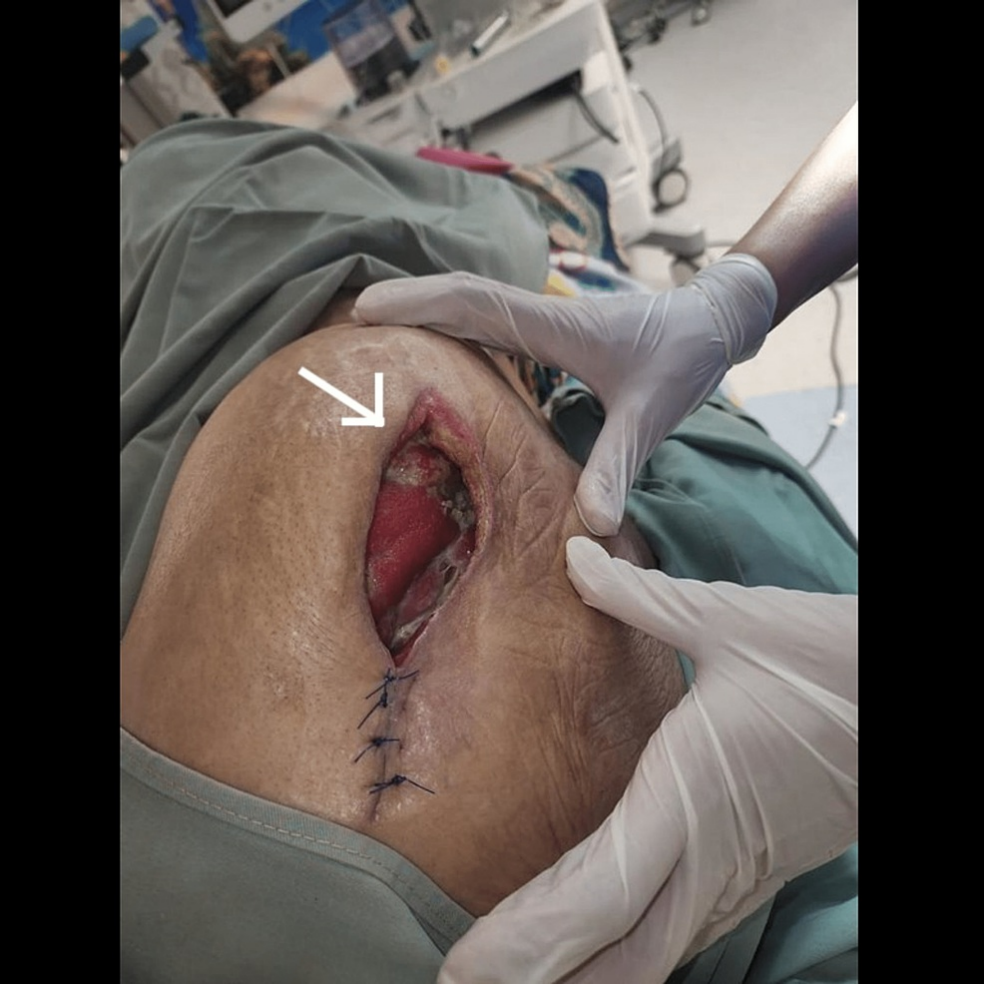
Wound at the time of discharge showing significant improvement

On the fourteenth day of admission and after seven days of negative pressure wound therapy, the wound dimensions were 14 centimeters in length and 4 centimeters in width. Wound edges were preserved and granulation tissue was present. No exudates were noticed and the perilesional area was normal (Figure 6). The patient was discharged from the hospital to home with the VAC dressing in place with a once-weekly follow-up.

At home, VAC dressing settings were set at the pressure of 125 mmHg and intermittent mode, i.e. 50 minutes on and 20 minutes off. VAC dressing was changed once weekly at every hospital follow-up visit. The patient tolerated VAC therapy comfortably.

**Figure 7:**
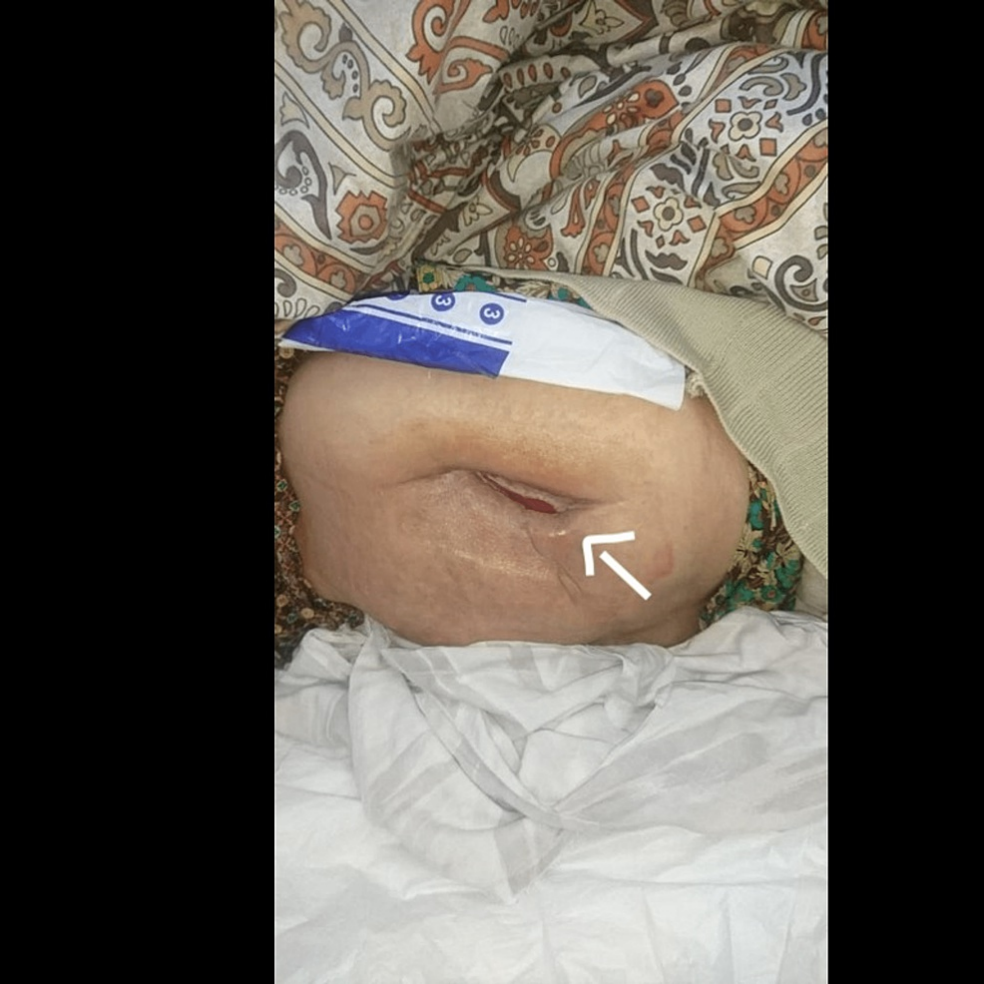
Wound condition after 35 days of treatment

On the third follow-up visit and after a total of 28 days of negative pressure wound therapy, a significant improvement in wound dimensions was noticed (Figure 7) and a decision was made to suspend the application of VAC dressing.

The conventional dressing method with humid saline gauze was used for the next three weeks till complete healing and closure of the wound were verified.

## Discussion

Necrotizing fasciitis is an infrequent but potentially lethal infection that quickly deteriorates soft tissue and fascia, leading to necrosis of the affected region [1]. The lower extremities are the most commonly affected area, followed by the abdomen and perineum. The gluteal region is a frequent injection site, and injections in this region carry a heightened risk of complications such as abscess formation and cellulitis [3]. Although rare, necrotizing fasciitis can occur following an intramuscular injection and has been documented in medical literature [3].

Group A Streptococcus is the most common cause of necrotizing fasciitis, but it can also be caused by other bacteria, including Staphylococcus aureus and Escherichia coli [1].

The initial management of necrotizing fasciitis involves immediate surgical debridement of the affected tissues [6]. The primary objective of surgical intervention is to eliminate all necrotic tissues, including muscle, fascia, and skin, to preserve healthy skin and achieve hemostasis [1]. The management of necrotizing fasciitis also involves administering broad-spectrum empirical antibiotics intravenously, considering the microbiological classification. In this case, linezolid combined with carbapenem and meropenem was selected as the empirical treatment and continued after the wound culture results yielded MRSA.

Postoperative wound care and proper nutritional support are critical for the patient's survival following surgical intervention for necrotizing fasciitis [4]. Conventional dressings have an enzymatic debriding action that is beneficial when used in conjunction with surgical debridement. The extent of tissue invasion associated with necrosis, the potential for severe septic systemic repercussions, and the presence of multiple underlying comorbidities in the patient serve as indicators of the severity of the condition. Thus, a comprehensive approach to postoperative wound management and nutritional support is essential for the patient's successful recovery [4].

Due to the persistent difficulty in achieving favorable outcomes in the management of NF wounds, negative pressure wound therapy (NPWT) has been proposed as an adjunctive treatment option [5]. NPWT has been shown to be effective in treating NF wounds, as well as complex wounds of other etiologies, such as burns, open fractures, fasciotomies, diabetic foot wounds, and pressure ulcers. Several studies have demonstrated the positive impact of NPWT in wound healing and infection control. For instance, a prospective study on 35 patients with NF treated with negative pressure wound therapy demonstrated a significant decrease in mortality when compared to conventional dressings [4].

NPWT provides a consistent negative pressure to the wound bed, which eliminates extravascular edema and enhances microcirculation blood supply and angiogenesis [4]. It promotes granulation tissue formation and reduces wound size and bacterial load. Despite these benefits, in some rare cases, the foam used in NPWT may lead to persistent infections in extensive wounds. Additionally, NPWT has some drawbacks such as inconvenience for ambulation, repeated dressing changes if required, difficulty in maintaining a hermetic seal, and pain and discomfort caused by suction. Nevertheless, the total cost of NPWT can be up to three times lower than traditional wound treatment for postoperative patients with acute treatment in the long term [5].

The application of NPWT can be used for various clinical objectives, such as serving as a temporary solution until definite surgical completion or facilitating wound closure by secondary intention [4]. The decision to use secondary intention wound healing was made due to various reasons such as the patient being unsuitable for surgery due to underlying comorbidities and the age of the patient. So negative pressure wound therapy was considered an effective solution.

Considering the expense, it is crucial to standardize the utilization of negative pressure wound therapy, focusing mainly on challenging wounds that are hard to manage, with the aim of enhancing the formation of granulation tissue and shortening the hospitalization period. Negative pressure wound therapy has been shown to be a secure and efficient option, leading to satisfactory scar formation, development of granulation tissue and epithelialization, closure of wound edges, and preservation of skin integrity around the wound, with minimal interference during dressing alterations.

## Conclusions

NF is a rare bacterial infection that can be very dramatic, with shock and damage to internal organs. Considering its severity and rapid spread, an early diagnosis is essential for speedy recovery along with serial debridement and adequate antibiotics. VAC application has helped achieve quick wound closure in some cases, especially those with co-morbidities like diabetes mellitus.
